# Dr. Beddoes's Collection of Papers on the Influenza

**Published:** 1803-09-01

**Authors:** 


THE
Medical and Physical Journal.

VOL. X.] SEPTEMBER 1, 1803. [NO. LV.

*Printed for R. PHILLIPS, by W. Thorne, Red Lion Court, Fleet Street, London.*

### DR. BEDDOES'S COLLECTION OF PAPERS ON THE INFLUENZA.

### PART II.

I Must begin with expressing my regret for the loss of a letter from Mr. MORRIS, Bristol; and another from Mr. HILL, Shepton Mallet.

The first (No. 30) expressed his belief that the influenza was not contagious, principally because he had known instances, where one or only a few of a family had been affected, notwithstanding free intercourse.

Mr. Hill (No. 31) inclined to the same opinion.

32. Dr. DOYLE, Ross, Ireland, July 17, 1803.

What they call influenza here is epidemic (I may say) every spring season. It has been particularly so for six or seven years past. It generally sets in with the cold dry weather in March, and varies with the degree of intensity of the vicissitudes of heat and cold until the middle or end of May. The latter was the case this year; we had some very warm days about the middle of April, shortly succeeded by very intense cold dry winds, until the end of May, during which month the epidemic seemed to be at its height here, becoming more mild as the heat increased in June, when it disappeared almost entirely.

It first made a noise by its appearance in large cities. May this not be owing to the great number assembled? Its appearance in Ross, a large country town, and in the country for twenty miles round, varied little, as also its decline. It was little mortal in any of its stages in Ross, particularly when early attended to, but was more mortal in the country; I do think much owing to the contrary circumstances and management.

The recovery of sound persons attended to was generally the 5th, 9th, or 11th day, and few relapses happened except in case of neglect, such were not violent; such as had the lungs before weak or affected, fared worse; and when death happened, which was not always the case even with them, it occurred about the 9th or 11th day.

Its sudden attack on whole families should induce me to think it was not contagious, but entirely owing to the then reigning constitution of the air, which was exceedingly sharp, and entirely originating from the general causes of diseases usually occurring in spring, varying in degree, and something in types, with those general causes and constitutions of the people. I could not find it communicated by clothes, further than by change in quantity or kind, which I believe sometimes procured it.

However, you must observe I am not fond of contagion, and verily believe it often exists but in the doctor's brain. In most cases of disease we cannot be at a loss for sufficient and satisfactory causes, which will excite similar disease in the same family, town, or even country, which I am certain it would be useless to recapitulate to you; and why should it not be the case in influenza, which (as far as my observation extends for some years) is chiefly of an inflammatory nature, varying to or from the real state of pulmonary inflammation, as we find in the different degrees of pleurisy, peripneumony, asthma, and other attacks on the lungs, which seems to be the organ principally, though not only affected. I have experienced, *in general,* the same plan of cure answer best, particularly when early applied, otherwise it did not answer as well as in the unequivocal pneumonia.

I said *in general,* as I think I have observed about five years past what is called influenza to partake much of an erysipelatous nature, the fever attending being much of that kind, and local attacks of erysipelas occurring exteriorly on many parts of the body, when the lungs were quickly relieved; I have not the smallest doubt but this type of disease often occurs unobserved, or is mistaken for real inflammation. In the former case bleeding did not answer except at the very commencement of the attack, and then moderate; it was destructive at any other stage.

You will observe it would far surpass the limits of this, to enter more at large on this subject; but as you have required I should say something of it, beg leave to offer you the above observations just as they occurred.

33. Mr. Hey, Leeds, July 20, 1803.

After mentioning his public and private occupations, observes, the influenza in slight cases so much resembled a common common catarrh, that no distinguishing criterion could be pointed out. But in general, the attacks of this epidemic were much more debilitating than in the common catarrh. Many complained of great soreness in the throat, when upon inspecting the fauces no morbid appearance could be perceived. A teazing cough was a general concomitant, though a few patients had all the common symptoms of the disease without cough.

Some felt great difficulty of respiration upon lying down, who were much relieved by leeches applied to the sternum, or the operation of an emetic. A frequent pulse and furred tongue generally attended, though many had the pulse betwixt 80 and 90, with a clean tongue, who were much otherwise indisposed.

A sudden transition took place in some, especially elderly people, from a febrile state to a state of debility. Stimulants, such as the volatile alkali, and spt. ætheris vitriolici, were of great service in such cases. If the disease was contagious, the influence of contagion must have been very rapid. But upon the whole, I should rather be inclined to call the disease epidemical than contagious.

34. Mr. Hill, Barnstaple, June 29, 1803.

The influenza appeared in this neighbourhood some time in February, and still continues to the present time.

The disease has not been general, but arose in some remote places about the same period, and in such still continues to shew itself.

In most instances, not above one or two in a family.

It was not contagious. In some families of ten or fifteen, not above one or two were affected. The same in villages.

It would be particularly flattering to convey to you some acceptable matter of observation; but so little have we seen of the disease in this neighbourhood, that I have little to offer. It has not been fatal in one instance within my knowledge, or that of my friend of South Molton, (Mr. Bryan) though we have heard of many cases; but, as far as we could trace them, they did not appear to us to have been cases of influenza. Every thing, from an aching tooth to a fractured leg, almost, has been deemed the influenza.

35. Mr. Goodwin, Wirksworth, Derbyshire, July 3, 1803.

I think the complaint first appeared in this place about the 20th of March, (I cannot be certain to two or three days) and almost totally disappeared by the middle of May. It was most general about the 10th of April. This country being very populous, and the intercourse almost constant, I had not an opportunity of observing whether there was any difference in the date of its appearance in more or less frequented places.

After it ceased to be general, there were some single cases, but these were few, and at no very distant period from the general affection. The complaint was so common about the 10th of April, that it seemed to me to be occasioned by some peculiarity of atmosphere, and not by passing from person to person; but of this I cannot pretend to be certain, and only mention it as matter of opinion. The disease sometimes occurred more than once in the same person; some attributed the return of the symptoms to what they called taking cold; to others, the recurrence was without any evident cause. The common symptoms were, frequent pulse, pain in the limbs and head, extremely troublesome cough, sickness at stomach, great dejection of spirits, and very great lassitude. In some cases the cough was incessant, night and day: these patients had also a great deal of fever. Some had considerable difficulty of breathing, and pain in different parts of the chest. In some the tonsil glands were enlarged and very florid; and I saw some cases where the inside of the cheeks, tongue, and throat were affected with aphthæ. The symptoms commonly abated, and sometimes went entirely off, in five or six days; sometimes in three or four days; and in some cases continued much longer.

When I was called in early in the complaint, I invariably gave an emetic. I never repented this practice, as it was, *in all cases*, attended with advantage. I then ordered my patients to drink plentifully of barley water and gum arabic, infusion of linseed, and to those who could bear it I gave small doses of James's powder or tartarized antimony. In cases attended with much pain about the chest I ordered blisters, and sometimes the loss of some blood. I soon learned that much bleeding was hurtful, by bringing on symptoms of debility. I heard of some people who were incautiously bled, who sunk under the disease. I did not lose one patient. The stomachs of many of my patients were much affected by even very small doses of the preparations of antimony, and some could not bear the least particle. In such cases, preparations of antimony were immediately abandoned, and very small doses of ipecacuanha substituted, and I thought with advantage.

When

When the mouth and throat were affected with aphthæ, I gave myrrh and bark, and with evident success. I allowed my patients to drink small beer, and a little wine diluted with much water.

36. Mr. Hodge, Sidmouth, July 4, 1803.

The influenza appeared in this neighbourhood about the middle of March, and ceased to be common about the same time in April, though after that time it now and then occurred; I have not seen many instances of its passing from one person to another, but I have seen many attacked in the same hour on the same day, and conceive it to be epidemic, resembling in many instances, particularly in old people, chronic catarrh; I have not been able to ascertain the situations least exempted from the disease. Am sorry my observations are so few; have only to add that it was not generally severe in my practice, not having lost a patient except when connected with peripneumonia vera.

37. Mr. Griffiths, Cardiff, April 9, 1803.

I am induced to believe the influenza is epidemic; because, in numerous instances, several of a family have been attacked on the same day.

In the majority of cases, the influenza is distinguishable from common catarrh.

Had not the influenza prevailed, the symptoms so much resembled, in several instances, common catarrh, that I should have been of opinion they belonged to the latter.

Generally the depression was more considerable than is common in catarrh; and the disorder had, in numerous instances, all the gradations from pneumonia to low fever.

The disorder so varied in its attack and progress, that I cannot, with any degree of correctness, specify the date of the disease.

38. Mr. Cooke, Gloucester, June 13, 1803.

The influenza appeared with us in February, and became less frequent in April, ceasing gradually, if not entirely, in my practice, through the last month.

I considered it an epidemic disease; I rather suspect it did not pass from person to person, unless it degenerated into typhus, which it frequently did from predisposition in the patient, from neglect, or from maltreatment. The above, I believe, consolidates complete reply to all your queries, to which I would add, that I always observed an attack of influenza immediately excite constitutional complaint (that is, those by which persons had been before frequently attacked) of whatever species they might be, whether rheumatism, nervous affections, paralysis, &c. and if there was a predisposition to phthisis, typhus, inflammation of any particular viscera, &c. these all occurred; but if influenza continued more than three or four days, symptoms of low fever always commenced, with reason to dread infection transferrable. The common characteristics were, fever, excessive perspiration, and little general pain, or universal pain without perspiration, accompanied with a peculiar noisy, disagreeable sensation, confined to one side of the head, nose, and face, (similar, I suspect, to the tic doloreux); this painful symptom would often baffle every attempt to remove it (after all other symptoms were dispersed) for, perhaps, two or three weeks, and then gradually or suddenly go off—sometimes terminating in abscesses in the frontal or nasal sinuses, abscesses in the ear, &c.

I have reason to think that the bladder in women was sometimes attacked with influenza, and that the eye and the œsophagus have also experienced the same affection without any material derangement of the general system in either case.

Emetics, diaphoretics, purgatives, and a generous, free dilution, was the plan I always found successful, and never observed the disease prove fatal, excepting when the chest was attacked violently, which was frequently the case with old asthmatic persons.

Your request of an immediate answer, compels me to send a rough sketch of my observations, which if of the least service to you, will very much gratify me.

39. Mr. Lomas, Aspatria, April 18, 1803.

As the disease has in general been so mild here, as to require little aid from medicine, I have had but few cases to attend to; I therefore must confess myself unable, as yet, to decide upon its contagious nature.

I have hitherto been accustomed to attribute the propagation of such complaints to an epidemic constitution of the atmosphere alone; but from instances which have recently occurred, I am rather inclined to think the present disorder contagious. The supposed mode of introduction into families; the succession of attack upon the individuals of those families; the sudden effect produced upon the system in twenty-four or thirty-six hours, after being exposed posed to it; some families being affected generally, and others at no great distance (in country situations) being perfectly free from it, are my principal reasons for suspecting its contagious nature.

Upon close examination, I think, in general, no doubt would arise, whether the case is catarrh or influenza.

Before the frequent occurrence of the complaint excited more strict attention, I think it probable that single instances might be mistaken for common catarrhal affections. But being myself affected towards the first in this neighbourhood so differently from what I had ever experienced in common catarrh, and afterwards hearing others describe what I had felt, the idea of a prevailing epidemic struck me before I heard any account of influenza.

In some of the cases I have attended, robust men complained, all at once, of an uncommon degree of languor, lassitude, muscular pain, and soreness; in some smart, in others scarcely perceptible, rigors preceded the more violent symptoms, such as oppression at the chest, pain in the head, &c.; these last generally took place on the second or third day, with greater and more sudden depression than I ever saw in common catarrh. In all there has been a great sensibility of external cold.

Although I have employed the lancet in two instances with good effect, yet, in general, I think the disease borders more upon low fever than pneumonia.

I cannot say that I saw a well-marked influenza before the 8th instant; it is now becoming daily more prevalent.

40. Mr. Lawrence, Cirencester, April 14, 1803.

I am not able yet to determine from my own observations, whether the influenza is contagious or otherwise. I think it may be communicated from one person to another, yet my observations lead me to consider its powers in that way to be weak.

I ground my opinion chiefly from having seen several instances where only one, and where only two, in families consisting of greater numbers have hitherto felt the disorder; some instances have occurred where two have slept together, and one only received it; this happened in my own family. Again I must observe, that it has appeared in families where the most, and likewise the whole, have felt it in succession.

In many cases I have remarked its great resemblance to the common catarrh; yet different constitutions seem to admit of considerable variety in the symptoms; in so much, that by comparing a number of cases, it may, I think, be distinguished from that disorder.

In some cases the symptoms so much resembled those of common catarrh, that I should have considered them such, had my mind been free from the idea of influenza.

Often the depression has been great and sudden beyond what is seen in common catarrh; gradations from pneumonia have been very frequent even down to feelings of great debility. I have seen no case shewing a disposition to degenerate into low fever.

The first case of well marked influenza I saw, I believe was on the fifth of March; it happened to be a robust healthy farmer, who the week before, on a journey into Essex, passed twice through London; he lives in a village about five miles from hence, where, at that period, the influenza had not appeared, but was then universal in London. Many cases come now daily before me, for it very much prevails at this time in this town, and in the neighbouring villages.

41. Mr. Custance, Kidderminster, June 13, 1803.

I need not trouble you with any particulars of the characteristic symptoms, as they are not only well known to you, from your own observation, but also from the different publications on the subject by various practitioners.

Whether the influenza has been a *contagious* disease or not, I really cannot satisfactorily determine. I am of Sir Roger de Coverley's opinion, when he so wisely decided, upon another occasion, that "much might be said on both sides;" however, upon the whole, I think that the preponderance of probability is in the scale of contagion. I will now state a few facts, that appear to me to favour this supposition. The influenza certainly gradually passed from one town to another. It prevailed at Stourbridge about a fortnight or three weeks before it was noticed at Kidderminster, and it was another fortnight before it appeared at Bewdley, which is three miles on the other side of us, so that it should seem to have travelled in a north-east to a south-west direction.

The first cases I saw of it, were three children, in one family, who sickened within a few days of each other; the father and mother both were attacked in about a fortnight after. The gentleman had it so severely that he did not recover from it in less than ten or eleven weeks. In my own family, my two servants were attacked together, and

I was

I was severely and suddenly seized about ten days after. My bowels were from the first very much affected, and the subsequent debility was considerable, and continued for a month or five weeks. My wife and child were so slightly affected, that it is doubtful whether they had it at all; and a sister of Mrs. Custance, who resides with us, certainly escaped altogether; but it very rarely happened that *part* of a family escaped. Many persons are persuaded that they were attacked repeatedly with it; but I think this a mistaken notion, and rather am of opinion that they had not completely recovered from the first attack, before some of the symptoms were aggravated by indiscretion and carelessness. Nevertheless, in one instance, I believe a child was seized a second time, a month or six weeks after she appeared quite well of the former attack. A gentleman, who had been confined to his room ten weeks with a broken leg, was free from the complaint during its rage amongst us, although many people daily visited him, (especially myself and other medical friends) yet he got about into the air only a few days before he shared the fate of his neighbours. The instances of mortality have been very few indeed amongst us. I lost three patients, one a girl of fourteen years old, the other two under a year; and I am persuaded that my medical brethren here were equally fortunate.

We considered and treated it as an inflammatory disease, with the precaution of bleeding very sparingly or not at all. Debility so soon supervened, that I always preferred blisters to bleeding; the latter was employed in only one case by me. The following mixture I found invariably useful after the first stage of the disease.

℞. Tinct. opii, gt. xx. spt. ætheris vitriol. ʒiij. infus. gent. c. ℥vj. ℔. to be taken according to the age of the patient.

I forgot to mention, that several individual cases of influenza have occurred since it ceased to be a general disorder.

42. Dr. Turton, Swansea, June 19, 1803.

Whatever information my memorandums or recollection can furnish you with, respecting this singular epidemic, I will with much pleasure communicate.

Its first appearance here was about the middle of March, and was preceded by sore throat and other diseases of debility. Its duration was not more than three weeks.

The general seizure did not appear to be different in date date here and in the country round. I saw two or three at some distance from hence before I was attacked myself.

Whether it was communicated from person to person, or whether the blow was generally produced from the same atmospheric cause, it is not very easy to determine.

It is however to be marked, that many families were not affected at all; and that when one of a family was seized, it seldom failed to extend its influence in a greater or less degree to most of the members of it.

The aged and infirm, and those who were from any cause much debilitated, suffered severely.

Of my patients only one died, and she had been previously bled before I saw her.

Those who persisted in the use of wine and good food were but little affected, and were soon restored. Children were principally afflicted in the eyes and lungs; women and puny persons with long continued catarrhs; men at the first attack had great confusion of intellect, probably from torpidity of the meninges of the brain.

The seeds of phthisical complaints were sown in some tender habits; and I am afraid its ultimate mischief is not yet vanquished.

The same causes, whatever they were, may again produce similar effects; it will therefore be desirable, whenever it happens, to have the collected opinions of men who personally and professionally marked its progress.

Epidemic diseases are too apt to appear and vanish without concern beyond the period of their existence, and with very little more than traditional memory of their relation, symptoms, and effects.

I rejoice exceedingly that you are about to register the history of this malady, which has certainly appeared before, and will doubtless appear again.

Did it not follow the line of the earthquake? and was not the earthquake some few years ago succeeded by a similar national affection?

43. Mr. Beaumont, Melbourne, Derbyshire, July 15, 1803.

1. The influenza appeared as early as the latter end of January, but became very general in February and March, and did not disappear till the latter end of May or the beginning of June.

2. As far as I can recollect, it appeared in a neighbouring hamlet before it appeared in Melbourne; this I am clear in, that it was much more general in that hamlet, before it was thus general in Melbourne.

3. We

3. We had single instances with us after it ceased to be general.

4. With respect to this query, whether it passed from person to person or not, I cannot so well answer. In many cases it appeared pretty clearly to do so; and again, in others, it was very difficult thus to trace it, it seemed rather to affect almost the whole family at once.

I shall just observe, that from what I have read and heard of the influenza, the inhabitants of this town and neighbourhood have been much more favourably dealt with, at least as to general attack and violence in individual cases, than many other places in the kingdom.

### 44. Mr. Pearse, Honiton, July 12, 1803.

1. The influenza appeared here the first week in March, and ceased about the middle of May.

2. It appeared in the neighbouring parishes at the same time that it did in this town.

3. By the end of March it was very general in the town and country, and continued so all through April and the first week in May, when it abated, and but a few cases appeared afterwards in a slighter degree.

With respect to your fourth query, whether it was propagated by contagion? Your superior judgment is better able to decide. The disorder was in many instances sudden in its attack; several were taken in the market, and came immediately to me for assistance; some whole families were seized with it, while, in other instances, two or three of a family would escape. I myself was taken down the second week in March; my daughter three days after my attack; a young man, my pupil, had it slightly near three weeks after, but not any one of the family had it besides, though constantly together.

Several whom I have attended, after being confined for three or four days, have got apparently well, but who have relapsed; the disorder has been more severe on them than the first seizure, and continued for seven or eight days. Nurses who have attended the sick, some have escaped, and some have had the disorder slightly.

### 45. Mr. Cambridge, Minchin Hampton, Gloucestershire, July 28, 1803.

The first appearance of influenza in this neighbourhood, which came to my knowledge, was on the 16th of February, ary; the last patient I attended, recovered the beginning of May.

In most instances, the disease, in its progress, was eight or ten days, though some continued ill several weeks, whilst others recovered in a few days.

After the epidemic became general and was on the decline, some who had not been previously affected were seized in a greater or less degree, and were longer recovering than those who were earlier affected.

From the observations I have been able to make on this disease, I remarked, that different constitutions were very differently affected. The most general symptoms I met with, were prostration of strength; a sudden and extreme debility of the whole system; an acute pain affecting chiefly the fore part of the head; a sense of stricture across the chest, as if bound round with a cord; a hard cough; a slow fever with a small weak pulse, and great depression of spirits. These symptoms were present, generally, the second day, accompanied with catarrh. I have met with many instances where the symptoms were widely different, varying in degree in different persons.

In others, the disorder manifested itself differently and betrayed symptoms of pneumonic inflammation; in some the pulse was quick and strong, beating an 100 strokes and upwards in the minute; great thrist, a dry foul tongue, with anxiety and nausea. Asthmatic patients suffered very severely, and some, I am informed, died from suffocation.

46. Dr. Frazer, Wisbich, Cambridgeshire, May 5, 1803.

The first case I saw of the influenza was in this town on the 11th of March, about which time it shewed itself in a great number of families. No instance of the disease (within my knowledge) had previously occurred in the circumjacent country; but in a few days, and before we could suppose the contagion could pass from one person to another, the whole neighbourhood for several miles, was affected. Persons living in most retired and isolated situations were attacked. It raged with great violence for a month in both town and country, when it began to decline.

From the 11th of April to the beginning of May, I have seen the disease occasionally, but it was no longer an epidemic, it appeared sporadically, and now at least not at all contagious. When this disease prevailed the most, and was attended by the worst symptoms, the weather was remarkably markably warm, the air dry and sultry, such as it is often observed in this country to be in July and August, previous to our sickly season, which follows in September and October. In the fenny parts of this county, and especially in the neighbouring parts of Lincolnshire, the influenza was soon complicated with or followed by a bilious remittent fever, of short duration, and often fatal termination. The sufferers were, in almost every instance, labouring poor, whose houses and beds were filthy, their diet scanty and of bad quality, who had little medical attendance, and who too often had recourse to spirituous liquors. The wealthy, on the contrary, in every case within my knowledge, escaped (i. e. recovered). Before I received your letter, I have often agitated within myself whether the influenza was propagated from person to person by contagion, or depended on a peculiar infectious state of the atmosphere. I have been much puzzled by what appeared to me contradictory evidence; but, upon the whole, I was inclined to refer the whole disease to the atmosphere. Several families, consisting of many individuals, were attacked nearly on the same day.

On the 16th of March, I was requested by a humane clergyman, as I rode along the road, to visit three poor cottagers families, who lived in a sequestered situation. In one family there were a man, two women, and four children, all of whom, together with the two other families, were attacked within twenty-four hours of each other. I have observed in many instances persons residing in the same family, and even sharing the same bed with the diseased, who have escaped; but I consider this fact as militating as much against the hypothesis of an infecting atmosphere as against the other opinion. My chief reason for supposing the disease to depend on the atmosphere was, from observing it in so many instances to be soon complicated with or followed by the prevailing diseases of the country, and which are not in themselves infectious. I think also that most of those who escaped were elderly persons or young children, who from their occupations or age were much confined to the house.

The measles and scarlatina, or any other infections disorder in this country, does not spread so rapidly, and embrace such a number of persons at the same time, as the influenza has done; besides they are diseases against which we are generally guarded, and endeavour as much as possible to avoid. If the disease has lately appeared to attack a single individual in a family where none were attacked before, before, and without infecting any other in the family; and if this fact has frequently occurred, as I think it has, does not this go far to prove the disease not contagious? But *suppose* it to be a fact, and it is equally if not more hostile to my hypothesis. Both sides of the question labour under great difficulties; the field of objections is too ample and difficult for me to enter on, and I must content myself with a few hints for your consideration. I have seen some instances where the disease in question seemed plainly to be communicated from the sick to the healthy, in others it gradually extended through a large family in a few days, while those who kept out of its way, have altogether escaped. I have been sometimes disposed to think, that the influenza was first produced by external causes existing in the atmosphere, and was afterwards propagated by human contagion. Is not the plague often produced and propagated in this way? and from what I have observed and seen of the yellow fever, I am disposed to think the same of it. I approve highly of your practice of fumigating with nitrous vapours, and have no doubt but it prevented the progress of the disease; but might not the same effects be produced, supposing the disease depended on atmosphere miasma? It at least corrected the atmosphere where those exposed passed most of their time. Your information respecting the re-sowing of barley is probably correct. I have not been in those parts of Norfolk where barley is sown, but have observed that the oats here have been much blighted by parching winds.

In a particular district of this country, a fever of a peculiar nature has prevailed, resembling the yellow fever in its symptoms, the rapidity of its progress, and fatal termination. I have often thought fumigations would be useful, but medical men are seldom at hand to direct them.

47. Dr. Frazer, Wisbich, Cambridgeshire, June 12, 1803.

The influenza, which, as I before informed you, ceased here about the middle of April, made its appearance again in May; the leading symptoms were the same as in the first attack. About the same time also a most malignant fever, having some symptoms in common with the influenza, began to rage in that part of Lincolnshire contiguous to us, which has proved fatal to hundreds.

I have myself been severely attacked, I think, by influenza, and confined to the house for the last twelve days; the the fever has now left me, but a troublesome cough, &c. still remain.

From this statement you will perceive that it was utterly impossible before now to indulge myself in the strong inclination I felt of writing to you. I find by the last number of the Medical and Physical Journal, that medical men are much divided in their opinions as to the causes of influenza. I perceive that you lean to that side which considers the disease in its origin and progress, as depending on human contagion. The arguments on this side I confess are strong and numerous, but I am less than ever inclined to subscribe to them, though I confess I am not able to refute them. In my last letter I informed you the disease appeared here in a great variety of persons, at the same time. The town of Wisbich had, at that period, a circumvallum of health; none of the neighbouring villages had been visited by influenza; and if the disease was imported here, why did it not affect our neighbours, through whom the infected persons must have brought it? *Very young* and *old* people here have generally escaped, and many, of all ages, in families where the disease severely afflicted others. I am aware that this last fact militates against my own theory, because it may be said, if the cause of the disease existed in the atmosphere, why were not all affected? I have scarcely any reply to this, but the old one *of different degrees of susceptibility*, and this reply is applicable to both sides of the question. But is it not only possible, but probable, that contagious particles may exist in the atmosphere, totally unblended and separated from its other component parts? and may not these particles be taken in by some, while others altogether escape? May they not also be taken in, in greater or less proportion? Many parts of England have not been visited by the influenza; how is this to be accounted for? Not surely by saying they had no communication with the diseased. I have seen the disease in the most sequestered situations. It may be worth while to inform you a little as to the country I now inhabit; it is a country *sui generis*. We have a few inconsiderable rivers moving sluggishly to the sea, but every four or five acres for twenty miles around me, is surrounded by a ditch with stagnated water. When these ditches are filled with water, the people are healthy, and, in proportion as water diminishes, our endemic diseases increase. We had very little rain here during the winter and spring, but much dry weather, and unusual warmth in the spring months. These are the reasons, in my my opinion why influenza was complicated with our endemics; and they lead me also to believe that influenza is a weed of our own growth, that is, that it would have appeared here without communication with any other place. What you observe with respect to the extending influence of miasmata is I think perfectly correct. It may be limited to half a mile or so, but we are not certain that that limitation depends upon miasmata being diluted by commixture with the atmosphere; there may be other causes. Some years back, while residing in Virginia, I visited a gentleman living in the county of Essex, upon the Rappahamac river. When this gentleman succeeded his father, he told me that he cut down a grove of trees that intercepted his view of the river, and also a marsh which lay near the river. Agues till then were unknown in his family. In the following and several succeeding years, almost the whole family, consisting of a number of Negroes and Whites, were attacked with intermittent fevers, and continued to be so until he replanted the trees, the removal of which Mr. Lomax at length suspected to be the cause of disease. Trees in that country are of rapid growth, and in a few years Mr. L. and his family were completely freed from the ague.

The re-appearance of the influenza after it had ceased for a fortnight is an argument with me for supposing the disease not contagious. In its second visit it did not affect several in a family as at first, but was confined to one, or at most two. I have seen a few cases in which the same person was twice attacked. Your patient, Mr. H. tells me, he had influenza at Clifton, and he certainly had it here.

Being struck with the re-appearance of the influenza, I took the liberty of questioning Dr. Frazer concerning the exact agreement of the first with the second complaint. In a letter, dated July 4, he says:

"I am certain the influenza re-appeared in this neighbourhood with all the general symptoms which first distinguished it. I have now two patients, whose cases are as compleatly marked as any that occurred to me in March."

48. Dr. Clark, Newcastle, Tyne, May 25, 1803.

I am most decidedly of opinion, that the influenza of this year spreads by contagion, although I have not been able to trace its introduction into this part of the country with that certainty I did in 1782. But as it appeared first in the sea-ports, and has, and is still, gradually extending itself into country villages, it appears probable that it was imported ported from London. It did not appear as an epidemic in this town till the beginning of the second week of March, and was at its height during the fine temperate weather in April.

Dr. Winterbottom gives me an account of some Negroes, from Sierra Leone, who have been apprentices to mechanics both here and at London, all of whom have had the influenza.

49. Mr. Barlow, Blackburn, May 27, 1803.

The enquiries you have stated respecting the nature of the influenza, I will endeavour to answer with as much precision as time and circumstances will admit, but not having had any prior intention of publishing my observations on the subject, you will consequently conceive that I am less prepared to convey my sentiments to you than I otherwise might have been. The existing difference between the influenza and common catarrh, appears to me to rest in the greater degree of febrile symptoms, resembling those of typhus, and the continued period to which the fever has in a greater number of cases been protracted in the former than in the latter disease. Some of the leading symptoms attendant on catarrh have been comparatively mild, and in many instances totally wanting in the influenza, such as sore throat, hoarseness, sneezing, and defluction from the eyes and mouth, when the other symptoms of a more malignant nature, as frequency of pulse, pains of the limbs and chest, extreme restlessness and anxiety, (and in a few instances of females, syncope) with great prostration of strength, and confusion of ideas amounting during the night to delirium, have marked the peculiarity of this epidemic, and shews in some degree the difference betwixt the two diseases, though in a majority of cases the leading characteristics have been nearly concomitant.

The influenza pervaded this town and neighbourhood about the beginning of April, and disappeared in about the space of six weeks; during this period the weather was milder than it generally is at that season of the year, and some part of the time the heat was greater than what is common in this district; yet this state of temperature produced no manifest change in arresting the progress of the disease, when in all probability, and under similar circumstances, catarrhal affection alone would have disappeared; and no difference was observable either in the appearance or disappearance of the disease, betwixt that of the town and adjacent country. With regard to the distinction between common catarrh and the influenza, as far as my observation goes, I am led to believe, that its progress in pervading the different districts of this island in succession, together with the diversity of symptoms above stated, render the two diseases in some degree different. To what this distinction is ascribable, whether to some specific state of the atmosphere, or susceptibility of constitution in particular classes of society of imbibing contagion, I am at a loss to determine; nevertheless, the disease has been comparatively less general amongst children than adults, and of much longer duration in the latter than the former; this circumstance however proves nothing, for we frequently observe diseases affecting children when adults are in a great measure exempted, such for example is the scarlatina.

I am well aware of the difficulty of establishing the truth respecting the contagious or non-contagious nature of the influenza; and with regard to its general diffusion amongst families where I have attended, I have not observed any material difference between the power of propagation of the influenza, and what has occurred at other times in the typhus fever. In many instances it has been diffussed through whole families; in others, though more rarely, the disease has only affected one or two individuals out of a number in the same family, and where they have slept in the same room. With respect to the fatality of the influenza in this town and its vicinity, I believe the cases of this description have been few; and out of a considerable number whom I have attended, only one terminated fatally; this was a person considerably advanced in life, and who had been long afflicted with an asthmatic affection. It will be unnecessary to particularize the mode of treatment in this disease, presuming that it is little different from the general practice adopted by others.

50. Dr. Bardsley, Manchester, June 15, 1803.

Your plan of prevention, by the means of separation and fumigation, appears to be best calculated, from the evidence of former experience in checking the spread of contagious fever, to obtain this desirable end. I have considerable reliance, from many and repeated trials, on the power of acid fumes to weaken the force of, if not altogether to destroy, febrile contagion; and I am glad to find, that fumigation succeeded in your attempts to stop the progress of influenza. I cannot say it was fairly tried in our House of Recovery, but it certainly formed a part of our customary preventive plan, at the time the nurses fell sick, sick, when attending upon the patients labouring under influenza: Yet as these nurses were not precluded from associating with the other domestic servants of the house, who were likewise afflicted with the disease, and concerning whom, no plan of fumigation or any other mode of prevention was put in practice; there ought not, perhaps, to be much stress laid on this point. The utility of separation is abundantly manifest. The facts, stated to have occurred in our lunatic hospital, shew this in the clearest point of view. But I proceed to answer your two queries.

I have lately seen more than a solitary instance of the occurrence of the epidemic, since it ceased to be general in this place, at the beginning of May. Other practitioners here have met with a few similar cases. I cannot affirm that the origin of the disorder, in these instances, arose from contagion being communicated through the medium of articles of dress or other families; but I think it not unlikely to be the case, as most of the members of the families, in which these solitary cases happened, had previously undergone the disease; and it was never thought necessary to adopt the usual precautions, (which all families, more or less, put in practice, after the existence of a contagious fever in their dwellings) to secure those who escaped the malady at an early period, from the danger of future infection.

The epidemic appeared in Manchester at an earlier date than in the neighbouring towns. An interval of about ten days elapsed from its spread here to the time of its reaching Bolton and some other populous places, situated at about twelve miles distance. It seemed to diverge from Manchester, as from a centre, to the surrounding country; but certainly appeared in the more crowded and populous towns, placed at the extremities of the circle, than in the intermediate space, which contains a thinner and more scattered population. This may be explained from the greater intercourse subsisting between the larger manufacturing towns and Manchester; and likewise from the consideration, that in towns, where the inhabitants are crowded together, the propagation of contagion is much more favoured than in less populous country villages and detached dwellings.

51. Dr. Redfearn, Lynn, Norfolk, May 23, 1803.

I have been favoured with your letter, requesting any information I could send you, relative to the late general epidemical catarrhal fever, or influenza.

From the result of my professional experience in this town and its vicinity during the prevalence of the above mentioned epidemic, there have occurred many corroborating proofs of its infectious nature. (viz.) One individual being seized out of five or six in a family, and all the rest having fallen ill in succession. Wherever this has happened, the origin of the disease might in my opinion, be satisfactorily traced to the first person affected. Yet, on the other hand, it must be acknowledged, that I have met with some few exceptions to this statement, and directly the reverse; as, where one or two in the same family have been attacked with strong symptoms of the influenza, and all the rest have escaped; but exceptions of this kind happen in all other febrile infectious disorders, nor do I know that this non-susceptibility. with respect to contagious impressions, can be referred to any particular age.

The influenza raged here in the warm weather, as it did in the other adjoining counties.

I do not know of any instance where it terminated fatally in this town, although few were exempted, more or less, from its attacks.

In reply to your enquiries, that in some parts of Norfolk the soil (naturally light of itself) has been made so loose by the dry winds, that the sown barley was laid bare, and that is was necessary to sow again; I have endeavoured to obtain the most accurate information from some respectable and intelligent farmers in different situations, and find that the case has actually been so; they think the atmosphere has been drier this year than it has been for some years past; and to this they attribute the effects of the wind on the barley-fields being so general.

52. Mr. Hadley, Derby, June 18, 1803.

The influenza began in this town about the beginning of March, and declined in a great degree about the latter end of April.

It prevailed at Ashbourne, in this county, about a week or ten days sooner than it did in this town, and I heard of a few cases in the north west part of the county still earlier.

Single cases occurr.d for some time after the disease ceased to be general; I have met with a few so lately as the latter end of May.

I am disposed to think the disease is not very infectious, for although I have seen several patients in the same family with the complaint, I have not been able to ascertain certain that those servants, and other parts of the family, who have slept with persons labouring under the influenza, have been more liable to it than such as have not been so immediately exposed.

Considering the disease is not very, if at all, infectious, I cannot say that I have known it spread from fomites.

In the treatment, I have found it necessary to bleed only six patients out of 200 or 300, and I have met with only one case which proved fatal, and that was in an old asthmatic patient; the disease in general yielded to the use of mercurial laxatives with antimonials, and the use of blisters, if the cough or pain in the chest were urgent. After the febrile state was removed, the debility and cough in many cases were very great, and which were very much relieved by the use of bitters and the digitalis.

53. Mr. Reddlesden, Ashbourne, Derbyshire, June 28, 1803.

In answer to your queries, I have to inform you, that the influenza appeared in this town the first week in March, and increased rapidly during the whole of that month, and began to decline toward the latter end of April.

It did not visit the neighbouring villages before the 18th of March; all these places have frequent communication with Ashbourne, being their market town.

Some few individuals were attacked with this disease after it had ceased to become general.

I have reason to believe it is strongly contagious, as scarcely one of the family escaped when it invaded a house.

As an example, among many others, I was employed in a school for young ladies, with a family of forty persons, none of whom, to the best of my knowledge, escaped the disease.

P. S. It may not be amiss to observe, that I heard it was at Derby before we had it.

54. Dr. Currie, Chester, July 19, 1803.

1. The first case of influenza I saw, occurred on the 30th of January. The disease gradually increased in frequency during the two first weeks of February; about the middle of the month it became very general, and continued to prevail till the middle of April, when its violence abated considerably. Cases, however, continued to occur during the remainder of this month, nearly, I think, in the same degree of frequency as at its commencement in the two first weeks of February. The last case I saw was that of a medical friend far advanced in life; he had been busily employed in attendance upon the sick during the whole of this epidemic, but escaped till the first of May, when, having been exposed to considerable fatigue from a long journey, and other circumstances, he was in the evening attacked with the influenza; some slight degree of hæmoptoe afterwards occurred, and he sunk under the disease with all the symptoms of peripneumonia notha. He had for some years had tender lungs, and been subject to catarrhal affections in the winter.

2. In several situations in our neighbourhood the influenza was later in its appearance than in Chester.

3 & 4. I consider the influenza as an infectious disease: this will appear from its progress in my own family. My youngest son, a boy, was attacked with the influenza on the 30th of January; his mother was seized with it upon the night of the 6th of February; my eldest daughter was taken ill upon the 15th of February; and upon the 23d and 24th of the same month, the whole remaining part of my family, twelve persons, were seized. On the 25th of February, the servants being rendered incapable of doing the business of the house, a woman from the neighbourhood was brought in to assist. She remained with us one week, and, on the 8th day from her entering my house, she was seized with the influenza. Her husband was taken ill upon the same day. It is necessary to observe, that she went home every night; this appears as if the husband was infected by fomites.

The following fact appears worthy of observation. At Holywell, a populous town in Flintshire, eighteen miles from Chester, and where there is a large cotton manufactory, a typhus fever of uncommon malignity had prevailed for a considerable time; the manufacturers and inhabitants of the town had not been free from it for more than two years. On the appearance of the influenza the typhus entirely ceased, and only one case of fever has occurred since. I have not for many years known this country so healthy as since the influenza disappeared.

65. Mr. Baddeley, Newport, Shropshire, June 27, 1803.

I know little of the influenza, except from my own and family's sufferings; seven out of nine of which were at one time, in different degrees, afflicted with it. I have been ill from it, more or less, since January last; my life was in great danger, and though I am much mended, I am not not yet well; every change of the wind, or the weather, renewing my hoarseness and cough, with alarming sensations of suffocation or choaking, which have distressed me from the beginning; the cough is truly spasmodic. My partner, who, from my confinement saw most of the cases, is, with me, of opinion, that it is infectious, as it went through most families where it began; though from the following circumstance we have reason to think, that with care and attention, it may have been prevented; for, in a school for ladies in this town, of near thirty boarders, two of the children had the influenza, but by carefully separating them from the rest, and keeping the fumes of Henry's vinegar constantly in the room, and about the bed, all the rest of the family escaped it; but this is the only instance of prevention I knew practised.

We think the influenza began to make its first appearance among us about the latter end of January last, and became most general in March, since when it abated; but I have seen a solitary case within this fortnight. It appeared to me that the east wind, or any portion of it, increased and aggravated the complaint.

We have not been able to discover any difference in the time of its commencing in this small town and the adjacent country.

We have no instance in our practice by which we can determine the communication of the disease by clothes, or such like.

56. Mr. Du Gard, Shrewsbury, April 18, 1803.

1. The first case that I perceived was on the 20th of February, in a little boy at the public grammar school in this town; he was seized with the symptoms of influenza, and was ill a week; his bedfellow was not infected by him, nor were any of the boys in the room, and there were twenty in the same apartment: nor did any one in the house become attacked with the disease till this boy had been well eleven days, at which time five or six were taken ill, and the same number daily, till four-fifths of the school were affected.

At a lady's boarding school the influenza first shewed itself on the eleventh of March, and in the course of the two and three following days between forty and fifty young ladies sickened. In a week they were all well again.

At a thread and linen manufactory, about a quarter of a mile from any part of the town, a house is appropriated for the dwelling of about two hundred apprentice girls.—

The influenza appeared there on the 16th of March, when six or eight were attacked. The disease continued eight or nine days, in which time not more than eighteen had it.

2. The influenza here has been generally accompanied with symptoms which denote inflammatory action.

3. About two-thirds of the patients that I have attended, have complained of an affection of the chest.

4. The pains of the limbs resemble much more those of synochus than those of acute rheumatism.

5. Most of the patients that I have seen, in all about 158, have had some disturbed action of the stomach and bowels; many have had diarrhœa, others vomiting, and some constipation.

6. In young subjects the complaint has generally gone off with very little expectoration. In the middle aged there has frequently been an inability to take a full inspiration; and many old people have died from suffocation, occasioned by the copious secretion of mucus in the chest, and the consequent want of due oxygenation of the blood. In some bad cases the patients have looked very livid for a few days, and yet have recovered.

7. The influenza appears to me to be infectious, and the contagion to operate in about twenty-four hours.

8. Some cases where the pneumonic inflammation has run high, blood-letting has been advantageously employed. In a few instances it has been necessary to repeat the operation, and in some elderly patients, the cake on the surface of the blood has curled in at the edges, quite as much as I ever remember to have seen it in pleurisy. In general, however, the disorder has yielded to the operations of calomel, antimony, and blisters, * without the aid of the lancet.

9. Gentle perspiration after an emetic was useful—profuse sweats kept up by heat were universally injurious.

10. Copious evacuations by stools, and the repeated employment of calomel, uniformly did good.

11. Opium given at first in large doses, or at all, seems to have done great mischief, which I have had opportunities of witnessing repeatedly. At the decline of the disease in elderly people, where the breathing has been quick

* Inflaming the skin very much with mustard cataplasms, has seemed to produce a better effect than blisters, and repeated when the oppression at the præcordia was great.

quick, five drops of laudanum with aq. kali, repeated every four or six hours, has been of great service. In these cases the short breathing I have supposed to be owing to debility and deficient absorption in the lungs.

About January, a great number of cats in Shrewsbury became seized with what is commonly called the *Hoost*, swelled heads, defluxion from the eyes and nose, with vomiting, sometimes purging, and sometimes costiveness. Some died, and others were relieved by means of opening medicines.

At the time the human species became a prey to the influenza, the dogs and horses were evidently affected: a few of the former have been seized with phrenitis, running about the streets biting every thing they met, and were killed as dogs labouring under the hydrophobia. Some persons who were bitten by one of these dogs, could not be satisfied till the part had been extirpated. I believe the disorder to have been simply phrenitis, because some dogs, so affected, have recovered, which favourable termination is supposed never to occur in hydrophobia.

Mr. Du Gard, Shrewsbury, May 9, 1803.

The instances of solitary cases of influenza are very rare. I have not in the course of my practice met with one, nor have any of the profession here; even supposing that there had been such cases, does it argue that the disease was not contagious?

The influenza was certainly sooner over in this town than in the villages around it.

During the Lent assizes the influenza was exceedingly prevalent in Shrewsbury: most of the country gentlemen composing the Grand Jury came to town in health, but very few returned without taking the disease along with them. At these assizes a cause was tried from Clun, twenty-seven miles S. W. of Shrewsbury; most of those who came here on that account were taken ill of the disease on their return, and spread it all over that little retired town.

Have you heard whether it was among the shipping?

The form of our jail is a quadrangle; the disease appeared in it on the 14th of March, and only thirteen prisoners had it out of about seventy, and all those were on the southern side of the prison.

In my former letter, I mentioned that a number of girls at a boarding school in this town had the complaint. I did not then inform you that it was also a day school, and that that those who lived in town, and came from infected houses, in all probability brought the disorder to the rest of their school-fellows. I have since learnt, that at another school, consisting *entirely of boarders,* not one of the young ladies (in all about twenty-six) had the complaint, owing probably to the care of the governess in not exposing them to infection.

I am told, from good authority, that at the Ketley Iron Works, whenever one person became affected with the influenza, it almost universally spread to all the people employed in that particular department.

It is the opinion of all our physicians and professional men, that the influenza is contagious.

Is it not possible, that when a town becomes filled with infectious matter, a calm atmosphere may, at times, be so far saturated with contagious poison, as to communicate the disorder, without contact, to habits susceptible of morbid action?

Might not light be thrown on the question of contagious and epidemic diseases, by investigating their progress in countries where there is little interior intercourse, compared with the incessant and universal communication through the different parts of this kingdom?

I cannot say that I have known it communicated by the clothes.

The influenza appeared at Welch-pool about the middle of March; it has been very prevalent, but in general not severe. Almost the whole of the Montgomeryshire militia quartered there have had it, and many are still ill.

The debility was the criterion by which I distinguished the influenza from common catarrh.

A cat was attacked with what I supposed to be the influenza; it was caressed by a little boy during the time of its illness, and the child became indisposed in about twenty-four hours; he was removed to another house, where he infected his two attendants in about the same time—these three cases were severe. The cat was costive, and by the advice of one of the family, had an injection: it was held by six people, five of whom were seized in the course of forty-eight hours with the influenza.—Is it probable that these people were infected by the cat, or was it only a coincidence?

Fumigation is employed to purify the air in the Salop infirmary morning and evening, and the floors are mopped with lime-water some hours previous to the first process; it did not, however, prevent the introduction of the influenza:

fluenza: indeed, how should it, when the friends of the sick are perpetually visiting them from the town?

53. Dr. Thorp, Ludlow, June 2, 1803.

The influenza made its appearance here about the latter end of February, and still continues, although in a trifling degree; it has been gradually declining for more than three weeks. It reigned with considerable violence for more than a week in Worcester, Birmingham, and Shrewsbury, before it made its appearance in Ludlow. Our situation might probably render us less liable to a disease of that nature, than many larger, closer built, and more populous towns; in addition to which I have observed, that those living in low situations have been first affected, and afterwards those on higher ground, and that it even now rages in some of the more mountainous parts of Wales with considerable violence.

It was seldom confined to one individual in a house, except where the acid fumes were used; but no regular certain day could be fixed as to the infectious stage of the disease (if infectious it is), as in some cases two or three were attacked on the same day; in four, five, or six days after another, and so on till generally; at least, often it went through the whole family; neither was having laboured under it once any security, as I know repeated instances of patients having been affected three or even four times, and the last attack (from the previous state of debility) has generally been most violent.

I do not recollect any instance where the disease appeared to have been communicated by articles of apparel.

Having answered your queries, I shall beg leave to state a few circumstances which have occurred to me.

1st. I almost invariably found, that where an emetic was given in the first, or early in the second day, the disease was cut short, and the patient after experiencing a slight degree of debility recovered.

2d. Wherever the lancet was used after the first day, the disease became tedious, debility excessive, almost constant nausea, pain in the head violent, and frequently delirium.

3d. Finding the disease spread rapidly, and that the common routine of practice only tended to abate instead of removing the distressing symptoms, and about this time the weather being close and sultry, I determined upon trying acid fumes, with a view of preventing, at least, the contagion spreading, if not of relieving my patient; in both both which cases it succeeded beyond my most sanguine expectations, as I found that it not only almost immediately proved highly grateful to the patient, but most effectually prevented the spreading of the disease in the family, as I do not, in any one instance, know of a person being infected after it had been made use of, and the cases where I recommended it were very numerous.

4th. Those drinking port wine in moderation, and living much in the open air, appeared less liable to infection.

5th. The weather, about the middle of April, proving hot, vomiting of blood was very common, and the disease, in many respects, put on a very putrid appearance.

6th. Weakly children and old people seem to have suffered most.

I have, as you requested, applied to most of the medical men, within 20 miles of this place. They all agree in this point, that no complaint was ever so prevalent, and that when the lancet was used late in the disease, it generally proved fatal. The measles have prevailed to a greater degree, in this part of the country, within these last two months, than ever was known, and elderly people, who had before escaped, have been very generally affected. I have had one patient of eighty-nine years of age, and great numbers of from fifty to seventy.

I have not heard of any practitioner in this part of the country using acid fumes.

59. Mr. Hall, Bridgnorth, July 1, 1803.

As accurately as I can ascertain, the first genuine case of influenza occurred on the 19th of March; from that time the disorder made rapid progress till the 28th, when we had the greatest quantity of disease. On that day I visited about seventy patients. On the 5th of April the disease put on a more inflammatory form, and I was induced to make pretty free use of the lancet for some days. After this the number of our patients was considerably lessened in the town; but we had still some sick families, at a distance, till the 26th, when the scene closed with us; the last case being as distinctly marked as the first.

Though at first of a contrary opinion, I was soon convinced that the disorder was of a very contagious nature. It appeared very clearly to me, and to the heads of all the numerous families where I attended, that the complaint did pass from person to person; and the proof is very sufficient, no one family having been affected *en masse*, where the disease did not first appear in some individual of it.

60. Mr.

60. Mr. Yonge, Sheffnal, April 27, 1803.

Just before the arrival of your printed letter I was confined to my bed by the disease which is the subject of it; and from the effects of which, I am not yet free. Grafted on my habitual catarrh, the pneumonic affection was severe, and after an illness of three weeks it left me extremely feeble both in body and mind, nor am I yet entirely free from the phthisical symptoms which at one time were considerable; *i. e.* I cough, (as in former times), on every alteration of exposure to warmer or colder atmospheres; and morning sweats, with some expectoration, still occur, more or less, daily. But I am certainly recovering both flesh, strength, and appetite, and doubt not shall soon be perfectly well. This cause, and my limited experience of the disease, disabled me from answering your queries, nor am I now able to do so, with much confidence in my own opinion. In reply to the first, my sentiments correspond with your own, that the disease is infectious, and for the following reasons.

2. In a great majority of those cases which have fallen under my own observation, I have been able to trace previous connection between the patient and some other person or place wherein the disease existed.

In several situations where the residents had little or no communication with infected persons or places, the influenza has appeared late, or not at all.

In both towns and families, the usual progress of the disease has been by such a succession of patients, as seem more naturally the result of contagion than of any cause operating more generally, such as may be ascribed to a specific state of the atmosphere. It has occurred, not unfrequently, that after one member of a family has sickened, all the others have followed in rapid succession; and sometimes three or four persons have, in the first instance, sickened at nearly the same time. In the only case of this kind I have met with, the previous connection of the patients with the infected was certain. I am unacquainted with any instance, where a village, town, or district, has become *at once* generally, or uniformly infected. On the contrary, from all the accounts I have been able to obtain, the progress of the influenza has been so slow and successive, as excludes the probability of any cause more general than contagion, or individual communication. I have not seen acid fumigations fairly tried.

3, 4, 5. Many instances have occurred where the influenza was so mild as not to be distinguished from common catarrh; catarrh; but, in general, it was distinctly marked, and easy to discriminate. I think, that in the present disease, the fever is much less dependent on, and less proportionate to the pneumonic affection, than in common catarrhal fever. In many cases the fever has been severe and permanent, where the affection of the lungs has been inconsiderable and soon relieved.

2d. The symptoms of fever are different from those which usually present themselves in pneumonia.

1st. In the excessive pain about the loins, hips, knees, and extremities.

2d. In the lassitude and uncommon prostration which accompany or quickly succeed the onset.

3d. The fever, which at first generally appears inflammatory, often degenerates into continued or low fever, nor will bear sometimes, even at the commencement, such treatment as in pneumonia is generally useful. I think I have observed also, both in children and adult patients, more affection about the larynx than is usual in catarrh, and which has produced the appearance of cynanche at the onset, and much aggravation of the cough in later stages of the disease. The first decided case of influenza I saw in the early part of March, but it had then existed in our neighbourhood near a fortnight, and has not yet ceased there.

61. Dr. Morrison, Wolverhampton, June 26, 1803.

I must confess I did not make a particular memorandum of the exact time of the appearance of the influenza; it was near Midsummer,* and made such rapid progress, that in a fortnight it arrived at its height, and continued in that state about six weeks, when its force gradually declined. There were several instances of the same person afflicted twice, and the last time in a more severe manner than the former; the old and weakly suffered most, and I have seen several instances in young and delicate females, where the greatest attention was necessary to arrest the alarming symptoms of an incipient phthisis. I did not find any material difference between the town and country as to the attack and continuation, but it certainly appeared first in the town. Notwithstanding there was a great variety in the thermometer during that period, I did not perceive the symptoms increase or decrease, in consequence of cold or heat.

---

* Should not this be Easter, or Lady Day? T. B.

heat. It is difficult to say whether it was caused from a certain constitution in the air, or passed from person to person, though I should incline to the latter opinion, because many in a family were seldom afflicted with this disorder, in the first instance, at the same time, but it regularly passed through a family, with the intermission of several days, and often weeks, from one person to another. I have no particular reason to ascribe it to fomites.

I wish you much success in your undertaking, as it will certainly be of great public utility.

62. Mr. Sandford and Mr. Field, Worcester. April 12, 1803.

1st. I am inclined to think the influenza *is* contagious, because I have observed that, for the most part, it affects persons in succession, living in the same house; and two persons sleeping together seldom or ever fail to communicate the disease. Mr. Field says, "this is a difficult question to decide." I am inclined to think it is *not*, because in some large families, where more than single individuals have been affected, it has not been in that regular gradation or period, that marks in general other contagious diseases. This answers the second query also.

3d. That at its *first* seizure, and if it was not known that such a disorder was prevailing, it would not be distinguished (in its first stage) with any peculiar or independent symptoms. This also answers the fourth query.

5th. In *all* the instances I have met with, the depression of strength, languor, &c. commenced in twenty-four hours, and sometimes sooner after the first attack. I have seen *less* of pneumonia in its progress, than of the disposition to low fevers; in some cases it has been accompanied with relaxation of the tonsils, and in some few the disease has terminated in periodical head-ach, and other characteristics of marked debility.

In answer to this question Mr. Field observes, "The symptoms of debility were unusually great and rapid, and in this instance chiefly was it distinguishable from common catarrh; in many it certainly degenerated into pneumonia and typhus."

6th. I do not recollect when I saw the first well marked case of influenza in this place, I will therefore transcribe Mr. Field's answer to this question, which is more decisive; and in the latter part, perfectly agrees with my almost daily experience.

"The

"The first distinct instance I saw in the neighbourhood of Worcester, was on the 10th of January, and the second not till the 11th or 12th of February. Fresh cases are now daily occurring, but I think the symptoms are not in general so violent as earlier in the disease."

Scarcely any invalid has escaped *this*, superadded to other complaints, but particularly such as have been labouring under affections of the chest.

63. Dr. SYMONDS, Hereford, June 30, 1803.

I perfectly agree with Drs. Blount and Hughes in every particular, excepting, that I firmly believe the disease *was contagious*; and how it was possible for any one to suppose it otherwise, I cannot conceive. In many cases in this neighbourhood, the attack was very similar to that of typhus fever; and so great was the pain in the head, and prostration of strength, that a few minutes produced delirium, and inability to move.

64. Mr. YEO and Mr. BURROUGHS, Clifton.

1. If the following facts, which have been collected from a very extensive practice, be at all conclusive, we think ourselves authorized to determine that this disease is certainly contagious.

2. First, what must have struck every observer, is the extension of this disease, for in whatever house it appeared, it almost invariably attacked every individual comprising the family, notwithstanding every precaution to guard them against the application of cold, and other causes commonly producing catarrh; and that the complaint appeared to be more prevalent with those using these means of defence, but who were within the influence of infection, than with those who were more frequently, *or* constantly exposed to the atmosphere, and yet out of the reach of contagion.

3. That it has made its appearance where the subject has not been exposed to the common air for several months, but with whom, others seized with this disorder have had frequent communication. Further, in a family, where the sick were so numerous that it became neceſsary to call in the assistance of a nurse, the woman, although in perfect health when she commenced her attendance, and during the same, was never exposed to the cold, yet took the complaint, and died.

4. In all cases where we have attended, we have found the the accession of the disease accompanied with more shivering, followed with more pyrexia, with a greater affection of the head; in the latter part of the disease, we have found it attended with delirium, and in almost every instance, with a greater degree of pneumonic inflammation than generally takes place in *common* catarrh.

4. Upon the whole, we think this a more violent kind of catarrh, but of a contagious nature; therefore, what has been enumerated in the third division, will appear sufficiently characteristic of this disease, to distinguish it from *common* catarrh.

5. In every instance, where the disease has appeared, even in the mildest manner, it was accompanied with greater debility than we ever knew attend almost any complaint of equal length; and we have it in our power to answer the inquiry, whether the disorder has not appeared in almost all the stages of pneumonia to low fever? by saying that we have a patient at this time, who was attacked with this disease three weeks ago, and who from violent pneumonic inflammation, will shortly die of phthisis pulmonalis and typhus fever.

From visiting houses where the disease had already existed, we have had opportunities of seeing patients on the first day of attack, and have seen others in the same family fall ill in succession; the febrile state for the most part terminates in the first week, unless attended with the more violent symptoms enumerated in the third division, and then it has continued a fortnight or more, and perhaps at length has ended in low fever; but this termination of the disease has occurred to us in two instances only. Although the fever generally subsides in a few days, yet the catarrhal symptoms continue much longer, and oftentimes remain very obstinate, insomuch that we have every reason to believe, that in the phthisically disposed it will be productive of consumption.

65. Mr. Taylor, April 10, 1803.

My observations of the late epidemic induces me to think it contagious.

My own family, with the addition of several others, gave the most decisive proofs of this.

The catarrh and influenza have their distinctive characters, which are easily observed.

The disease did not terminate as catarrh usually does, but left a train of low nervous symptoms, with universal debility and total relaxation.

The first person I visited in the complaint, was on the 10th of March; the last, about the end of the month.

66. Mr. Tudor, Bath.

I am disposed to think it contagious, (though not at any considerable distance) and for the reasons which I shall state in the answer to the next query.

I have generally observed, that as soon as one individual of a family became infected by it, most or all the others soon took the infection; and some cases occurred to me where the person had not, for some months past, been exposed to the atmosphere.

In many cases with great difficulty. In general, however, it is characterized by great depression of strength and spirits.

Mild cases were scarcely distinguishable from common catarrh; but besides the great languor immediately succeeding the febrile stage, the cough I think was peculiar in having a hollow sound; in some cases, almost resembling the hooping cough.

The former part of this question I have already answered in the affirmative; and the different gradations of the complaint were so great, that cases existing in the same house, at the same time, were not always sufficiently characterised to bear the same name.

67. Mr. Bond, Glastonbury, July 15, 1803.

Agreeably to your request, I will endeavour to answer your queries as circumstantially as possible.

1st. The influenza appeared here the beginning of March, became general the latter part of that month, continued so through April and part of May, began to decline about the middle of that month, and finally left us early in June.

2d. I did not remark that it had different appearances or effects in different villages in our vicinity except to the upper part of this town (the highest situation in this neighbourhood) which escaped its influence.

3d. After the disease was general, the instances that occurred assumed an intermittent type.

4th. It appeared to me to be contagious, as it did not seize families all at once; for, first one individual was attacked, and in a few days after more were, and so on until all, susceptible of the disease, had been affected by it. Children under the age of ten years, almost universally escaped its influence.

The

The general symptoms of the epidemic were, first, chillness and shivering, alternating with flushes of heat; spontaneous languor and debility were the grand characteristics; transient flying pains of the head, breast, shoulders, back, knees, &c. succeeded until the pain fixed on some part more particularly, when it became very distressing; and in all, the mucous membrane lining the mouth, lungs, nose, &c. were more or less affected; in the lungs it produced a tickling cough, attended with soreness of the breast, and expectoration of thin, sharp mucus; others had the throat more affected; and in others, the eyes were the organs most affected; in some an intolerable head ach was the most distressing symptom. The pulse was generally weak and quick, from 100 to 120 in a minute; the tongue moist, and covered with a white mucous; the urine high coloured and clear. Those who had a predisposition to pulmonary complaints suffered severely, as did those who were afflicted with dyspepsia; in fact, all those who died might be referred to one or other of those classes. There were two or three instances of dyspeptics who were carried off by it, even before their friends thought them in so much danger as to require medical assistance; they seemed, from what I could learn afterwards, to have been carried off from excessive debility. The general remedies made use of, when the disease was pure and unmixed, were pediluvia, neutralized ammonia, sp. æther. nitr. wheys, cool air, &c. and as I remarked that those who had angry eruptions uniformly recovered, it led me to the use of blisters (which were of great service); opiates, if had recourse to when the cough was incessant and attended with a thin defluxion from the lungs, were found of great benefit. Though no friend to phlebotomy in this disease, yet particular instances occurred, *in the young and robust*, where the lungs were much affected, that made it absolutely necessary; in one instance, to the extent of three times in 36 hours; and I believe the patient owes his life to the free use of the lancet. The high degree of inflammation in this case arose from his getting wet through two or three times on the day that he was first attacked.

The dyspeptic patients labouring under the disease, were obliged to be treated in a different manner. The contents of the stomach, &c. were obliged to be evacuated by gentle emetics, and doses of calomel and jalap, and afterwards the vis vitæ was assiduously supported. When the disease assumed the intermitting type, recourse was had to red bark as soon as the intermission was compleat, and if taken to the extent of half an ounce in sixteen hours, never failed to prevent the recurrence of the fit.

68. Mr. Symes, Bridgewater, April 9, 1803.

Having been absent from Bridgewater the greatest part of the months of February and March, I have not had it in my power to make such observations on the prevailing influenza, so as to answer your questions with any degree of accuracy.

The influenza had not made its appearance at this place on the 12th of February, at which time a considerable number of the inhabitants went to London, where many of them were immediately seized with the disorder; and as far as I can learn from the report of others, it did not appear at Bridgewater, until the return of some of the people from London, who either then were or had been affected with it. There are many places in the neighbourhood of Bridgewater with which the inhabitants have no intercourse, where the influenza has not yet made its appearance.

August 4, 1803, Mr. Symes, in conversation, assured the Editor of these papers, that from inquiry among his medical brethren, he ascertained the exactness of the above statement.

69. Mr. Steel, Abergavenny, July 6, 1803.

1. The first person that applied to me was seized with influenza in the market on March 14. As the attack was sudden, and the symptoms violent, she had great difficulty in getting home; she appeared, when I saw her, to labour under violent spasmodic asthma. I had no fresh case after April 20.

2. The disease did not appear in the country before the 20th of March; near this time I observed it at Bleanavon iron works, where are about 1200 people.

3. In some situations I believe that infection was communicated from the sick to the healthy.

At the iron works above mentioned, the epidemic was particularly violent, and in several instances fatal to *young men;* in these cases the patients were carried off by typhus fever. They were first attacked with pain in the head and back, *and had no symptoms of thoracic inflammation;* indeed I know of no young man dying who had pulmonary disease, except it existed before.

I observed also, that if a person was attacked in a house where another died, or where the complaint terminated in typhus, typhus, the symptoms were more severe than where the complaint had not (may I say) degenerated to that state.

Whether influenza was contagious in its common form or not, I am convinced it was when it terminated in typhus fever.

4. I did not observe the men attacked in classes at Bleanavon; but those employed about the fire were generally sufferers from pneumonia, and the colliers and miners from pain in the back, &c.

Every woman I attended in child-bed was greatly distressed with cough and fever.

A gentleman who had been in the north of England, came home perfectly well, when his family were ill of the complaint; in less than three hours after he went into the house, he was taken ill in the same manner as his wife and servants.

70. Mr. Reece, Cardiff, June 11, 1803.

With regard to my opinion, if influenza be contagious, I answer: I firmly believe it is contagious, though I also believe a particular state of atmosphere or a particular disposition in the habit, or both, first produced it, and might continue to produce it a short time, till the wind changed from and to every point of the compass, which it did during the reign of influenza. Surely *then* influenza was kept up by contagion. I have known many in a family ill, and some in a very slight degree in comparison to the others. It began here, as far as I know professionally, on February 18. Sometimes only one in a family was affected, sometimes it began with one, and ran through the whole house. I have had five or six, or even more in one house, where the mistress first had it, her sister next, the waiting maid, the nurse, the footman, the groom, house maid, and cook. The husband (who drinks full enough) had it in so slight a degree as to require no assistance. I do not recollect any person ill within six weeks ago. In my midnight midwifery gossipings, I made many inquiries of the old women who attended them. The constant result has been, "It came in the air to one person in the village, and then most of us who went to see her or him caught it." I asked if they found it more catching when the sick person was confined to a close room; but I could get little satisfaction in that inquiry, as in this country the doors of the cottagers fit the frames so badly that there is plenty of air coming in; and the windows, as I have often experienced to my sorrow, have admitted too much, from being gene-

 rally rally in a shattered condition. As to the treatment I used, (and here I feel myself on the brink of a precipice) I generally gave a saline mixture in a state of effervescence with emetic tartar, sometimes with camphor, opium, emetics or laxatives, according to circumstances. I do not recollect any thing further, but will feel happy in answering any questions you shall put.

71. Mr. Williams, Bridge-end, Glamorganshire, April 11, 1803.

As far as I am enabled to judge from my own observations, I am undoubtedly of opinion, that the prevailing disorder, which has been generally termed influenza or catarrhal fever, is in its nature truly contagious, and communicable, like the poison of typhus, measles, or small-pox, either in a gaseous state or in the form of infectious effluvia.

That the changes of the atmosphere have a very great influence over the human system is undoubted; that these variations combined with moisture have a tendency to produce catarrhal and pneumonic disorders is equally certain; but why a whole family should experience the effects of a disorder which a neighbouring one has not at all suffered from, appears to me inexplicable upon any other principle of reasoning, than that whatever might have been the cause or wherever the origin of the disorder, its increase was owing to the diffusion of a poison and the active operation of some kind of morbific particles. This could not have been the case had it merely depended on the variations either in the weight or temperature of the atmosphere, for why should an effect be partial, and at different times produced, where the influence of a cause had been contemporaneously and universally operating? I have known an instance where several persons (who had lived in a situation, which had remained free from the disorder) upon leaving it, and passing through a country and mixing with people among whom the epidemic was very rife, return with all the marked symptoms of the influenza. From circumstances, I am convinced they could not all have been affected with cold, and, excepting the prevailing epidemic, the country was healthful. How then could this have happened, unless that in one situation they subjected themselves to exposure to some contagious influence? If you suppose a marked predisposition in one person to catarrh from the slighest cause, it can scarcely be deemed probable probable that the same cause could have operated generally upon several others. These are the grounds I form my opinion upon.

I think a general comparison of cases enables us to distinguish between the two diseases, though undoubtedly in many instances, the commencement, the pyrexia, coryza, want of appetite, as well as the symptoms of amendment, (where an increase and freedom of expectoration seem to have removed the disorder, or perhaps as frequently a diaphoresis) have bore so striking a resemblance to those generally produced by catarrh, as scarcely to allow the nosologist reason and opportunity for discriminating.

Had I seen a solitary case only, I would have denominated the disease catarrh, though here I should be at a loss to account for appearances in some degree anomalous; but far from conceiving the disorder to have originated from any infectious miasma, I would refer the protraction of it to something idiopathic in the constitution of my patient, or suppose nature might have been thwarted in her operations from terminating catarrh in the usual manner. In elderly people, I have observed a considerable suffusion of bile, so much so, that the tunica conjunctiva of the eye has been nearly as much coloured as in jaundice. In such cases I should attribute the slow progress towards recovery to something wrong in the hepatic system and its secretions.

It is under this head I should form my diagnosis, for scarcely in any catarrh have I observed so great and so immediate a depression, or the pain in the back and limbs so violent; and at the decline of the disorder, the lassitude, the total incapacity for exertion, with the great disposition to profuse perspiration from the slightest cause, appear to me to distinguish the influenza from catarrh. The loss of appetite, even after the removal of all febrile symptoms, was very marked and particular, and I have observed that a week or nearly a fortnight elapsed ere the stomach became reconciled to usual habits of diet. I have seen many cases where pneumonia accompanied the catarrhal fever, and indeed others where cordials and a generous regimen seemed to have proved highly beneficial towards removing the disorder.

The first case I saw was about the beginning of the second week in March, and the last attack on the third of April.

[ To be continued. ]

